# The approach to managing perinatal anxiety: A mini-review

**DOI:** 10.3389/fpsyt.2022.1022459

**Published:** 2022-12-15

**Authors:** Victoria Anne Silverwood, Laurna Bullock, Katrina Turner, Carolyn A. Chew-Graham, Tom Kingstone

**Affiliations:** ^1^School of Medicine, Keele University, Staffordshire, United Kingdom; ^2^Centre of Academic Primary Health Care, Bristol Medical School, University of Bristol, Bristol, United Kingdom; ^3^Midlands Partnership NHS Foundation Trust, Trust Headquarters, St George's Hospital, Stafford, United Kingdom; ^4^Applied Research Collaboration (ARC) West Midlands, Keele University, Staffordshire, United Kingdom

**Keywords:** perinatal anxiety, perinatal mental health, mini review, maternal mental health, perinatal care

## Abstract

Perinatal Anxiety (PNA) is defined as anxiety occurring during pregnancy and up to 12 months post-partum and is estimated to affect up to 20% of women. Risk factors for PNA are multiple and can be classed as psychological, social and biological. PNA negatively impacts on the mother, child and family. PNA is not well-recognized and diagnosis of PNA can be challenging for clinicians. There is currently no validated case-finding or diagnostic test available for PNA. PNA has been less extensively researched than perinatal depression (PND). Clinical guidance currently recommends pharmacological and psychological therapies for the management of women with PNA, however the limited research available suggests that other intervention types may also be effective with some evidence on the effectiveness of non-pharmacological interventions in primary care for PNA. This article provides a mini-review of PNA, summarizing current evidence around PNA including risk factors, the impact of PNA, the process of diagnosis of PNA and focussing predominantly on available management options for PNA.

## Introduction

Perinatal anxiety (PNA) is anxiety that occurs in the postnatal period and up to 12 months after birth (1). This mini-review presents an overview of the latest evidence on PNA, focusing on treatment options but also providing a review of current literature around PNA in relation to etiology, impact and screening. It will discuss PNA within the context of Perinatal Mental Health (PMH) problems, and describe risk factors for PNA. Following this, the review will outline the impact of PNA on women, their family and society and discuss challenges with diagnosis of PNA. Finally, it will discuss the potential, currently available intervention options which could help to manage or alleviate PNA. In comparison to Perinatal Depression (PND), the literature around PNA is less well-established and less well-understood, therefore this mini-review contributes toward addressing this current evidence gap (2, 3).

## Methods

Systematic search strategies were conducted across 11 healthcare related databases (including EMBASE, Medline, CINAHL and AMED) utilizing keywords relating to PNA as well as relevant MESH terms. A literature review was synthesized from papers describing the epidemiology and etiology of PNA, the diagnosis and management of PNA as well as relevant clinical guidelines such as The National Institute for Health Care and Excellence (NICE) Clinical Guideline 192: Antenatal and Postnatal Mental Health (CG192) (4).

## Discussion

### Perinatal anxiety (PNA) in the context of perinatal mental health (PMH) problems

Global incidence of all Perinatal Mental Health (PMH) problems in women is estimated to be between 12.8 and 20%, (5) however a recent meta-analysis estimated the international prevalence of PNA could be as high as 20.7% (6). This means PNA is likely to be a more common PMH condition than Perinatal Depression (PND), which is reported to affect around 11.9% of women in the perinatal period (7). PNA may occur alone or alongside other PMH problems, for example PNA is often comorbid with PND (8). PMH problems contribute to significant levels of morbidity and mortality in the UK. Currently, the leading cause of maternal death following the 12 months after birth is suicide, which can be preceded by psychosis, PND or PNA (9).

It was recognized, prior to the COVID-19 pandemic and associated restrictions, that there is an urgent need to increase current funding and expand available PMH support. This was reflected in the NHS Long Term Plan (10), with a promised increase in funding. This appears increasingly important in light of the negative impact of the COVID-19 pandemic on PMH services worldwide with closure of community spaces and reduced access to social support (11) and it has been announced that PMH hubs will be established across the UK which aim to address these issues (12). In general, it is recommended that a multi-disciplinary approach to the management of women with PMH problems should be utilized, with services across primary and secondary care working together to provide appropriate and timely access to services for women with PMH problems (2).

### Risk factors for PNA

Risk factors for PNA are multiple and can be classed as psychological, social or biological (13). They include lack of partner or social support, history of abuse or domestic violence, personal history of mental illness, unwanted or unplanned pregnancy, adverse life events, high perceived stress, present/past pregnancy complications, pregnancy loss (14) and having a medically complex pregnancy (15). The neurobiology of PNA is not well-defined and appears to be heterogeneous and dependent on individuals (16). PNA symptoms can range from mild to severe and include excessive worrying, physical symptoms (such as palpitations, sweating and shakiness), social avoidance (17, 18), rumination over distressing thoughts [such as their baby coming to harm (19)] and nocturnal vigilance where mothers are unable to sleep for fear that their children might stop breathing during the night (20).

### Impact of PNA

Evidence around the effects of PNA on the mother and her developing child is inconsistent (21). PNA has been linked to neuroendocrine changes in the maternal brain (22), altered neurodevelopment of fetus and child (23–25), increased rate of adverse pregnancy outcomes such as pre-term birth or low birth weight (26–28), and risk of ongoing maternal mental health problems (4, 28). PNA has also been suggested as a risk factor for paternal anxiety and depression in the perinatal period (29, 30). It has also been reported that women with PNA may struggle to identify appropriate ways to seek medical support, and may access emergency medical care at a greater frequency than those without PNA (31).

Along with the potential adverse consequences for individuals and their families, PNA can also affect wider society. It has been estimated that the annual financial cost of PNA amounts to £20,794 per woman and £14,017 per child over a 10 year period when taking into account increased use of public services, loss of quality adjusted life years (QALYs) and productivity. (32) For children, as much as 70% of these costs are due to the increased risk of childhood disorders, such as behavioral or development disorders (33).

### Screening for PNA

It has been suggested that diagnosing PND is the widely accepted PMH priority in the perinatal period, meaning that other PMH problems such as PNA may be missed and therefore are underdiagnosed and undertreated (34). This appears to be changing though, with awareness of PNA amongst HCPs becoming more common (35). Case-finding for PND seems to be more acceptable using tools such as the Edinburgh Postnatal Depression Screening (EPDS) tool (6, 36) and the Patient Health Questionnaire 2 (PHQ-2) (4). In comparison, although NICE guidance recommends that Healthcare Professionals (HCPs) use the Generalized Anxiety Disorder 2 (GAD-2) case-finding tool (which has been validated for assessing GAD but not specifically in PNA), the use of GAD-2 is less widely seen, thus PNA is under-recognized and under-treated. It has been suggested that the GAD-2 might not be appropriate to use at antenatal appointments, as it is not specific enough to the experience of pregnant women (37). In some instances HCPs might try to use aspects of EPDS to assess PNA as it has been suggested that the EPDS is sufficiently ‘bi-dimensional' in order to be able to accurately screen for PNA by several studies (38, 39). However, there is some disagreement within the literature around this with critics of the EPDS suggesting that as it was originally designed to case find for PND it misses some of the nuances of PNA diagnosis, and is not able to adequately differentiate between symptoms of PND and PNA (40).

Currently there is no validated tool to aid diagnosis of PNA, but two specific screening tools have been developed recently to try and improve PNA diagnosis. The first is the Perinatal Anxiety Screening Scale (PASS) which is a 31 item self-report questionnaire that measures the following areas: general worry and specific fears, perfectionism, control and trauma, social anxiety and acute anxiety and adjustment over the past month (18, 41). The second is the Postpartum Specific Anxiety Scale, which is a 51 item measure specifically for postpartum anxiety detection (42). To date, neither of these have been validated and approved in the UK so are not included in UK clinical guidance.

Multiple challenges to diagnosing and managing PNA are detailed within the literature. In the UK, women are only offered a few routine scheduled appointments with midwives within the perinatal period and have no planned antenatal care with any other healthcare professionals (43). This means that identification of PNA is often opportunistic and may rely on women self-identifying that they have symptoms, consulting a HCP and discussing them (44, 45). Several studies report that women may be reluctant to disclose symptoms of PNA and might intentionally decide not to discuss them with HCPs (46–48). It has been reported that some women who have disclosed and discussed symptoms of PNA with a HCP felt their concerns were dismissed and did not receive sufficient or appropriate care support following this (3, 49).

### Interventions for women with PNA

NICE guidance suggests that treatment options for PNA include both pharmacological and psychological based therapies or a combination of the two (4). This guidance identified a gap in the evidence specifically around non-pharmacological management options for PNA and has recommended that further research is needed in order to develop psychological interventions to identify and treat moderate to severe PNA in particular (4).

The remainder of this mini-review will focus on discussing the current evidence-base for the range of interventions available to manage women with PNA.

### Pharmacological therapy

Antidepressant medication is recommended as a treatment option for moderate-severe PNA (4). Selected Serotonin Reuptake Inhibitors (SSRIs) are the most commonly prescribed medication option (50), and the SSRI sertraline is generally considered to have the lowest risk profile (45, 51) It has been recognized that there is considerably more established evidence for treating depression in the general population than in a perinatal population (52) and there is less evidence to support the use of SSRIs for PNA compared to PND (53, 54) Similarly to guidance around Generalized Anxiety Disorder (GAD) (55), the use of benzodiazepine medication is not routinely recommended unless experiencing “severe anxiety and/or agitation (4).”

The safety of medication prescribed to manage mood and anxiety disorders during pregnancy and whilst breastfeeding has received substantial attention, but there is a lack of consensus about safety. Several studies report links between taking medication and complications such as hypertensive disease in the mother during pregnancy (56), low birth-weight (57), and neurodevelopmental problems (58, 59). In contrast, two recent meta-analyses conclude that there is insufficient evidence to confirm that perinatal medication usage is associated with any significant harm to the developing fetus or breastfeeding child, but cannot confirm that there are no risks, suggesting that further research is required (52, 60).

The uncertainty around the safety of taking antidepressants during pregnancy results in women reporting decisional conflict about the potential risk of harm to their children weighed against the potential benefit for improved mood or reduced anxiety in themselves (61, 62). Inconsistent advice from HCPs around the use of prescribed medication in pregnancy adds to their confusion and highlights potential knowledge gaps for some HCPs. (49) These concerns place greater emphasis on the use of non-pharmacological therapy options (63) to be made available for women.

### Psychological therapies

Both women and HCPs view psychological therapies as an acceptable intervention for PNA (44, 45, 64, 65) and these therapies are recommended by NICE for the treatment of PNA (4). However, whilst there is established evidence that therapies, such as Cognitive Behavioral Therapy (CBT) and Interpersonal Therapy (IPT), are beneficial in the general population for managing symptoms of generalized anxiety disorder (GAD), there is less evidence that confirms their benefit in the perinatal population (33).

Several systematic reviews have considered the impact of psychological therapies on PNA (65–67) and suggest that whilst psychological therapies appear to be effective in managing symptoms of PNA, there is a limited number of high quality randomized control trials (RCTs) from which to draw these conclusions. In comparison, the evidence seems to be more defined for PND where several systematic reviews support the use of psychological therapies for treating PND, including CBT (68) and ITP (69). A study utilizing Behavioral Activation (BA) techniques for PND concluded that it might also be beneficial for PNA but this has not been formally studied (70).

Two studies which evaluated the effectiveness of psychological therapies to treat PNA include “CALM Pregnancy,” (71) and “MUMentum,” (72). “CALM Pregnancy” was a pilot study which investigated the effectiveness of mindfulness based CBT for PNA delivered in a group setting; the study explored women's perspectives of the intervention. The authors concluded that participants demonstrated statistically significant improvements in their PNA symptoms when measured using the Beck Anxiety Inventory (BAI) (73) and provided positive feedback about the intervention, which was encouraging and suggested further, larger randomized controlled trials (RCTs) would be helpful to confirm these findings. “MUMentum Pregnancy” investigated an internet-delivered CBT programme for PNA and concluded that whilst their findings suggested that the programme was effective, further RCTs are needed to establish this (72).

Several systematic reviews have considered the effectiveness of electronic/digital or internet-based psychological therapies (72, 74–76) to manage PNA. It has been suggested that electronic/digital health resources have the potential to reach many women who require support for PNA problems and improve accessibility to treatments (77). Critics of internet-based therapies argue that currently available technology requires further development (78), as the currently available online resources for PNA are of variable technological quality, with a limited evidence-base and many have not been formally evaluated (79).

It is currently unclear whether psychological support delivered by trained professionals is superior to less resource intensive options, such as guided self-help for PNA (80, 81). This is an area which also requires further exploration.

### Mind-body interventions

Mind-body interventions evaluated for their effectiveness in managing women with PNA include relaxation therapies, such as listening to music and guided imagery, and mindfulness based physical activity such as yoga or Thai Chi (67). Most of the existing evidence around mind-body interventions is based around antenatal anxiety specifically, meaning there is limited evidence for their use in the postpartum period.

Several small pilot studies have suggested that antenatal mindfulness-based interventions, such as mindfulness-based CBT and (71) yoga (82) might be of benefit as they demonstrated promising results for managing PNA with high participant satisfaction (83–85). Mindfulness-based family education has also been suggested to be a potentially helpful option for some women and their families (86, 87) when utilized as an approach by the whole family unit.

The effectiveness of yoga on anxiety in the antenatal period has been evaluated in several studies across the world in various different healthcare settings and is an effective management option for PNA (82, 88–92) Importantly, women's views of yoga in pregnancy are positive, with women experiencing PNA reporting that it has helped them to manage worry and anxiety and is generally considered to be a low risk activity when delivered by trained yoga teachers (93). Yoga has also been demonstrated to lower salivary cortisol levels in pregnant women, suggesting that it has a physiological effect on the body as well as the mind (91, 92).

The use of guided imagery (94) and relaxation therapies (95–97) within pregnancy to manage PNA has been investigated by several studies however there is a lack of evidence about the effectiveness of these techniques during the postpartum period. Relaxation therapies have been proposed to be more helpful if women are taught the techniques by trained therapists (97).

Several studies based in China (98–101) have investigated music therapy for women with PNA and suggest it is an effective way to reduce anxiety and improve mood in women with PNA. A systematic review and meta-analysis which assessed the overall effectiveness of music therapy on PNA agreed, however, it noted that the overall methodological quality of the studies included was weak (102). It is also important to consider if the results from this could be generalizable to populations outside of the countries where the original studies were conducted. Studies have been conducted in the UK and Europe to assess the use of music to treat anxiety around and during childbirth (103, 104) but there is no current evidence to support the use of music therapy in PNA specifically, demonstrating another potential gap in the current literature.

### Supportive interventions

Whereas, PND is a reasonably well-understood condition amongst HCPs (105, 106), in comparison, HCPs may feel unsure about how best to manage women with PNA (44). Women's experiences of the support they received from HCPs for PNA varies (64), indicating that greater awareness and understanding of PNA amongst HCPs would be helpful.

There is limited research around peer and social support as interventions for women with PNA, although there is evidence from several small pilot studies that suggest group interventions involving psychological therapies such as CBT or IPT might be effective (107–111). It has been noted that in a larger scale trial, where the impact of peer support and group IPT for women with PND, found that the interventions lowered anxiety levels as well as depression scores, which is promising (112). Group settings for psychological therapies may also offer a cost-effective option where resources are limited (71). It has been suggested that peer support may exacerbate PNA if not delivered and supported appropriately (109).

Lack of partner support has been identified as a risk factor for developing PNA (113). Currently, there is insufficient evidence to establish whether interventions aimed at improving partner support could alleviate PNA symptoms (114, 115).

### Alternative therapies

Whilst alternative and complementary therapies are not widely recommended by NICE as a treatment for PNA due to insufficient evidence (116), international studies support their use. Techniques used more frequently outside of the UK, Europe and the USA which may be of benefit, include acupuncture and acupressure (117), aromatherapy (118) and massage therapy (119). There is some evidence to support the use of massage therapy in the UK, but further positive evidence is needed before it could be clinically recommended as a treatment option (119).

## Conclusion

This mini-review summarizes the breadth of current literature around PNA. It provides a review of PNA, primarily from a UK perspective and is not intended to be exhaustive of all available PNA literature. PNA affects a significant proportion of women worldwide and has multiple risk factors. Diagnosis of PNA can be challenging and currently there is no validated screening or diagnostic tools available specifically for PNA in the UK.

The currently recommended treatments for PNA are pharmacological or psychological (4). As this review demonstrates, there are other interventions that have the potential to be helpful for women with PNA, but there is insufficient quality evidence to support their formal recommendation in clinical guidance, therefore further primary studies exploring interventions are needed. As experiences of PNA are so individualized, an individualized approach to management might be beneficial in order to support women with PNA (120). The profile of PNA needs to continue to be raised amongst women and HCPs in order to increase awareness of the condition.

In recent years more literature specifically focused on PNA has been published, increasing our understanding of the condition. Research gaps exist in several areas, particularly around the use of peer support, and the uncertainty about which interventions are the most effective for treating women with PNA. In order for amendments to be made to clinical policy and guidance, future research needs to focus on the diagnostic and management journey of women with PNA in order to improve their experiences and decrease the impact of PNA on individuals, their children and their families.

## Author contributions

VS drafted the manuscript with contributions from all authors. All authors reviewed the final manuscript.
